# Different diagnostic performance of plasma fibrinogen and D-dimer in periprosthetic joint infection: a propensity score matched study

**DOI:** 10.1186/s12891-021-04282-w

**Published:** 2021-05-07

**Authors:** Xi Chen, Wenwei Qian, Xisheng Weng, Jin Lin, Jin Jin, Yiou Wang, Shibai Zhu

**Affiliations:** Department of Orthopedic Surgery, Peking Union Medical College Hospital, Peking Union Medical College, Chinese Academy of Medical Science, Beijing, China

**Keywords:** Periprosthetic joint infection, Fibrinogen, D-dimer

## Abstract

**Background:**

Fibrinogen (Fbg) and D-dimer have been used as biomarkers for the diagnosis of periprosthetic joint infection (PJI). However, previous research has reported conflicting results on the diagnostic value of D-dimer in comparison to Fbg, C-reactive protein (CRP), and erythrocyte sedimentation rate (ESR).

**Aim:**

This study aimed to: (1) determine the optimal threshold of plasma Fbg and D-dimer in the diagnosis of PJI and compare their diagnostic value to that of CRP and ESR; and (2) investigate whether Fbg and D-dimer perform differently than CRP and ESR as diagnostic indicators for different types of PJI.

**Methods:**

A total of 115 revision cases after total hip arthroplasty (THA) and total knee arthroplasty (TKA) were identified. Based on demographic characteristics, 25 culture-positive cases were matched to 50 culture-negative cases using propensity score matching. Sensitivity, specificity, receiver operating characteristics (ROC), negative predictive value (NPV), and positive predictive value (PPV) were calculated and compared.

**Results:**

The optimal thresholds were 2.72 mg/L for D-dimer, 3.655 g/L for Fbg, 12.64 mg/L for CRP, and 27 mm/h for ESR. Levels of plasma Fbg, D-dimer, CRP, and ESR were significantly higher in the culture-positive group than the culture-negative group. Fbg, D-dimer, CRP, and ESR showed sensitivity of 0.92, 0.56, 0.92, and 0.88, respectively, and showed specificity of 0.84, 0.96, 0.94, and 0.80, respectively. The ROC curve showed that CRP has the highest area under the curve (AUC) (0.94), followed by Fbg (0.90), ESR (0.87), and D-dimer (0.81).

**Conclusions:**

Plasma Fbg exhibited a similar diagnostic performance compared to CRP and ESR in predicting culture-positive results in PJI. Plasma D-dimer showed high specificity but low sensitivity. In our study, Fbg and D-dimer did not show better diagnostic performance with different pathogens and different types of PJI. Further studies are required to investigate the difference between serum D-dimer and plasma D-dimer in the arthroplasty population.

## Article summary

### Article focus

1. Determine the optimal threshold of plasma Fbg, D-dimer, CRP, and ESR in the diagnosis of culture-positive PJI in revision arthroplasty.
2. Compare the diagnostic value of Fbg and D-dimer to that of CRP and ESR in PJI and explore potential causes for any observed differences.
3. Investigate whether Fbg and D-dimer perform differently than CRP and ESR in diagnosing different types of culture-positive PJI and associated pathogens.

### Key messages

1. Plasma Fbg exhibited similar diagnostic performance in PJI compared to CRP and ESR.
2. Plasma D-dimer is of limited diagnostic value in PJI.
3. Further studies are required to investigate the differences in diagnostic performance between serum D-dimer and plasma D-dimer in the arthroplasty population.

### Strengths/Limitations

1. Propensity score matching was utilized to eliminate bias caused by confounding factors (age, sex, BMI, and comorbidity). Another benefit of matching is to gain more efficiency and reduce bias in studies with a small sample size.
2. Sample size was limited in our study.
3. There were significantly more knee cases in the PJI group than in the Aseptic group, which may have led to potential bias.

## Background

The diagnosis of periprosthetic joint infection (PJI) has been challenging for decades. Current diagnostic criteria include physical findings, culture, histological analysis, and serological/synovial fluid biomarkers [1, 2]. However, physical signs are sometimes subtle in patients with PJI. Histological analysis is unavailable until after surgery. Leukocyte count and other markers from synovial fluid have good diagnostic value. However, it can be difficult to acquire a sufficient amount of joint fluid for a culture or other tests [3], and venous blood samples are more easily accessible. Erythrocyte sedimentation rate (ESR) and C-reactive protein (CRP) are essential serological markers in the diagnosis of PJI. However, ESR and CRP values can be normal in cases where PJI is caused by a low-virulence organism [4, 5]. Therefore, the use of more accessible biomarkers would improve efficiency and accuracy in the diagnosis of PJI, especially if they exhibit good diagnostic performance in chronic and low-virulence infections.

Novel biomarkers including D-dimer, fibrinogen (Fbg), alpha-defensin, and interleukin-6 (IL-6) have been introduced to improve the diagnostic criteria of PJI [6]. D-dimer and Fbg are routinely tested in most medical facilities. Parvizi et al. [1] introduced D-dimer in the 2018 Musculoskeletal Infection Society (MSIS) diagnostic criteria, in which it plays an equal role to that of CRP. However, there are conflicting research results on the diagnostic value of D-dimer in comparison to Fbg, CRP, and ESR, in part because different optimal diagnostic thresholds have been utilized [3, 7–9]. Shahi et al. [3] reported a high diagnostic value for D-dimer. Li et al. [8] reported that D-dimer has limited diagnostic value in PJI, while Fbg showed promising diagnostic value. Limited evidence has been found on whether these biomarkers are valuable in recognizing infections caused by low-virulence pathogens and chronic infections.

Therefore, this study aims to: (1) determine the optimal threshold of plasma Fbg and D-dimer in the diagnosis of PJI and compare their diagnostic value to that of CRP and ESR; and (2) investigate whether Fbg and D-dimer perform differently than CRP and ESR as diagnostic indicators for different types of PJI.

## Methods

Approval from the Institutional Ethics Committee was obtained. We retrospectively reviewed patient admission records from December 2012 to March 2020. Patients admitted for total knee arthroplasty revision and total hip arthroplasty revision were recorded. Patients were excluded if they: (1) underwent revision surgery for dislocation and acute periprosthetic fracture; or (2) underwent second stage revision (reimplantation) surgery following PJI. Patients were then divided into culture-positive cases and culture-negative cases based on culture results.

Propensity score matching was used to match each culture-positive case to culture-negative cases. The following covariates were matched: (1) age; (2) sex; (3) height; (4) weight; (5) BMI; and (6) comorbidities, including coagulation disorder, auto-immune disease and malignancy, or taking anticoagulation medication or immunosuppressants. Four culture-positive cases were excluded due to unsuccessful matching. Eventually, 25 culture positive cases were matched to 50 culture-negative cases.

Blood samples were taken 1–3 days following admission and prior to the day of surgery. Plasma D-dimer, Fbg, CRP, and ESR were tested among a series of preoperative lab draws. Pre-operative joint aspirations were taken when PJI was suspected. Results of physical examination, culture, and time of PJI symptomatic onset were recorded. All cases were followed up at three to six months post-operatively in outpatient clinic.

Statistical analyses were conducted with SPSS 25. Propensity score matching was conducted using STATA 14. The ROC curve was generated by Graphpad 8.0. Continuous variables were analyzed by unpaired *t* test and results were recorded as means and standard deviations. Dichotomous variables were analyzed by chi-square test and recorded as frequencies and ratios. A P-value less than 0.05 was considered statistically significant. The sensitivity, specificity, positive predictive value (PPV), and negative predictive value (NPV) of each biomarker were calculated and recorded with 95 % confidence intervals (95 % CI). Positive predictive value is the probability that a patient with a positive test result has the disease. Negative predictive value is the probability that a patient with a negative test result does not have the disease. The Youden index was used to determine the optimal threshold for D-dimer and Fbg. A receiver operating characteristics (ROC) curve was generated, and we used its corresponding area under the curve (AUC) and 95 % CI to determine the diagnostic value of each biomarker.

## Results

The demographic characteristics of included cases are listed in Table 1. There was no statistical difference in terms of sex, age, BMI, and comorbidities between the two groups. There was a significantly higher proportion of TKA cases in the culture-positive group than in the culture-negative group. The levels of plasma D-dimer, Fbg, CRP, and ESR were significantly higher in the culture-positive group than in the culture-negative group (Table 1).

**Table 1:** Comparison of demographic characteristics and biomarkers between groups

	Culture-positive (*n* = 25)		Culture-negative (*n* = 50)		*P*-Value
Age (yrs)	60.88 ± 14.21		65.24 ± 9.71		0.176
Sex					0.307
Male	10		14		
Female	15		36		
BMI	26.15 ± 4.08		25.17 ± 3.83		0.325
Hip/Knee					0.007
Hip	6		29		
Knee	19		21		
Comorbidity^a^	10		14		0.307
D-dimer (mg/L)	3.96 ± 3.14		1.19 ± 0.91		< 0.001
Fbg (g/L)	4.75 ± 1.34		3.08 ± 0.60		< 0.001
CRP (mg/L)	61.31 + 63.93		5.09 ± 5.95		< 0.001
ESR (mm/h)	56.88 ± 31.89		19.70 ± 16.58		< 0.001

Comorbidity^a^ Patients with coagulation disorder, autoimmune disease, other infections or malignancy, or those taking anti-coagulation medication or immunosuppressors

**Fig. 1:**
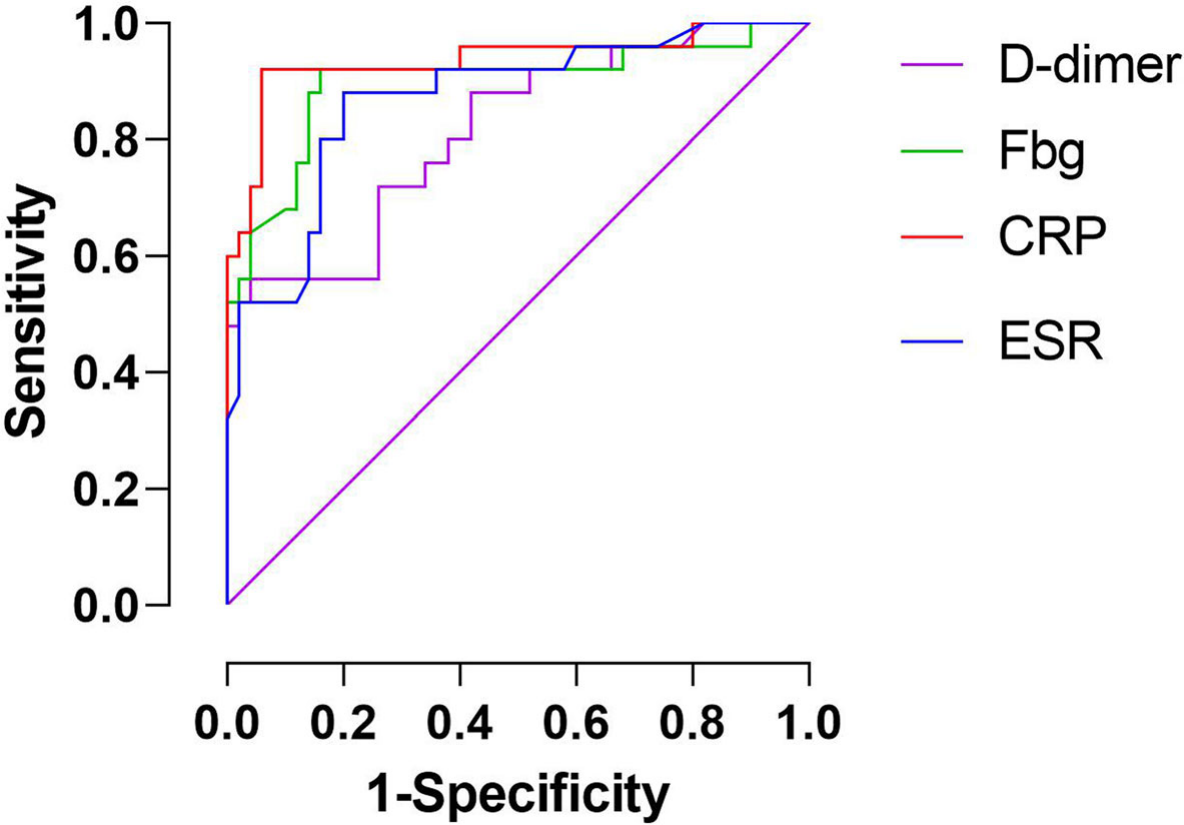
ROC Curve

The optimal threshold of D-dimer and Fbg was determined based on the Youden Index. The optimal thresholds were determined to be 2.72 mg/L for D-dimer and 3.655 g/L for Fbg. The ROC curve showed that CRP had the highest AUC (0.94), followed by Fbg (0.90), ESR (0.87), and D-dimer (0.) (Fig. 1). CRP and Fbg had the highest sensitivity of 0.92, followed by ESR (0.88). The sensitivity of D-dimer was 0.56. D-dimer showed the highest specificity (0.96), followed by CRP (0.94), Fbg (0.84), and ESR (0.80). The positive predictive value for CRP, Fbg, ESR, and D-dimer were 0.89, 0.75, 0.69, and 0.74, respectively. The negative predictive value for CRP, Fbg, ESR, and D-dimer were 0.96, 0.91, 0.93, and 0.95, respectively. The diagnostic performance of Fbg, D-dimer, CRP, and ESR is listed in Table 2.

**Table 2:** Diagnostic performance of each biomarker

	Threshold	Sensitivity	Specificity	AUC (95 % CI)	PPV	NPV
Fbg (g/L)	3.655	0.92	0.84	0.90 (0.808–0.987)	0.758	0.912
D-dimer (mg/L)	2.72	0.56	0.96	0.81 (0.710–0.919)	0.741	0.955
CRP (mg/L)	12.64	0.92	0.94	0.93 (0.866–0.999)	0.885	0.959
ESR (mm/h)	27	0.88	0.80	0.87 (0.781–0.957)	0.687	0.930

*PPV* positive predictive value, *NPV* negative predictive value

Nine cases were defined as acute PJI (diagnosis within 3 months after primary implantation) and they were culture-positive. Sixteen cases were defined as chronic PJI. For acute PJI, the positive rates of Fbg, D-dimer, CRP, and ESR were 88.9 %, 66.7 %, 100 %, and 88.9 %, respectively. For chronic PJI, the positive rates of Fbg, D-dimer, CRP, and ESR were 93.8 %, 50 %, 87.5 %, and 87.5 %, respectively. Coagulase-negative *Staphylococcus* was identified in nine cases and coagulase-positive *Staphylococcus* was identified in four cases. The types of infection and the responsible pathogens are listed in Table 3.

**Table 3:** Types of PJI and identified pathogens

	Acute (*n* = 9)	Chronic (*n* = 16)	Total (*n* = 25)
Positive culture	9	16	25
Coagulase-negative *Staphylococcus*			
* Staphylococcus epidermidis*	0	8	8
* Staphylococcus capitis*	1	0	1
Coagulase-positive *Staphylococcus*			
* Staphylococcus aureus*	2	2	4
*Streptococcus*			
* Streptococcus mitis*	0	1	1
* Streptococcus pneumoniae*	1	0	1
* Streptococcus agalactiae*	1	0	1
* Streptococcus anginosus*	0	1	1
* Escherichia coli*	1	2	3
* Brucella*	1	0	1
* Salmonella*	1	0	1
* Klebsiella pneumoniae*	0	1	1
* Bacillus fragilis*	0	1	1
* Corynebacterium rhizogenes*	1	0	1

## Discussion

Fibrinogen and D-dimer are by-products of the process of fibrin clot breakdown. Increased levels of both Fbg and D-dimer represent the activation of the coagulation process [10]. Researchers have examined the relationship between the processes of coagulation and inflammation. Coagulation biomarkers have pro-inflammatory effects, as fibrin mediates the inflammatory process and D-dimer promotes neutrophil and monocyte activation. Persistent inflammation also contributes to hypercoagulable state [11, 12].

To date, most studies that have focused on the diagnostic value of D-dimer and Fbg have utilized the MSIS criteria [9, 13–15]. However, the MSIS criteria have been called into question by researchers and is susceptible to subjectivity among clinicians, which could introduce selection bias. Therefore, culture result was used as the primary objective outcome measurement in this study. This allowed us to analyze CRP and ESR as well since they are not interpreted as part of a gold standard test for PJI.

Based on the AUC of each biomarker, Fbg (0.90), CRP (0.93), and ESR (0.87) exhibited similar diagnostic value, while D-dimer (0.81) exhibited a comparatively poor diagnostic value. The sensitivity of D-dimer was much lower than that of Fbg, CRP, and ESR. Previous researchers have reported conflicting results on the role of D-dimer in the diagnosis of PJI. A meta-analysis published in 2020 by Liu et al. [16] found good diagnostic performance of Fbg and moderate diagnostic performance of D-dimer. Our previous systematic review concluded that serum D-dimer is of comparable diagnostic value to CRP and ESR for PJI, while in this series, plasma D-dimer was of limited diagnostic value [17]. One explanation for these differences is that serum and plasma D-dimer exhibit different diagnostic performance. In serum samples, D-dimer is measured after standardized coagulation. Cross-linked fibrin degradation products appear in the blood before standardized coagulation and are mostly included in serum samples after coagulation. The fibrin degradation products remaining in the serum sample adds to the measured serum D-dimer, which may result in a higher level of serum D-dimer comparing to plasma D-dimer. However, the effect of residual fibrin degradation products in the serum has not been quantified [18–20]. Paniccia et al. [18] found serum D-dimer was significantly higher than plasma D-dimer in the majority of pregnant women and they found no correlation between serum D-dimer and plasma D-dimer. To our knowledge, there is no study reporting the difference between serum and plasma D-dimer in arthroplasty populations. Contemporary researchers have reported on the limited diagnostic value of plasma D-dimer and conflicting results regarding D-dimer. Two studies [8, 15] have shown the limited diagnostic value of plasma D-dimer compared to CRP and ESR. Shahi et al. [3] reported promising results of serum D-dimer, while Pannu et al. [21] and Huang et al. [7] reported limited diagnostic value of serum D-dimer. Differing testing methods and samples were used in different hospitals, which may have influenced our clinical interpretation of these studies. As reported by Liu et al. [16], multiple methods can be used to assess fibrinogen and other biomarkers. Further studies in this field are required to investigate the diagnostic value of these biomarkers with different testing methods and samples.

Racial difference is considered one of the reasons for the differential diagnostic value of D-dimer across different studies. However, based on the data from previous research, studies published in the United States of America reported controversial outcomes on the diagnostic value of D-dimer [3, 21], as did studies published in China [7, 8, 14, 15].

Plasma Fbg showed comparable diagnostic performance in PJI compared to CRP and ESR. Our results are inconsistent with the results from Li et al. [8]. Fibrinogen has been found to play several key roles in host antimicrobial defense. Fibrinogen can limit the growth and dissemination of bacteria within infected tissue and can support the activation of host immune cells [22]. In peritoneal infection, fibrinogen can contain *Staphylococcus aureus* and other pathogens [23].

As a coagulation biomarker, Fbg is superior to D-dimer in the diagnosis of PJI. One possible explanation might be that Fbg and D-dimer are associated with the infection process by different mechanisms. Another reason might be that D-dimer has shown good diagnostic value in detecting deep vein thrombosis, pulmonary embolism and other inflammatory diseases [24]. However, limited evidence has been reported regarding the diagnostic performance of Fbg in these conditions [25]. Patients with thrombosis and inflammatory diseases were included in our study, which may have influenced the diagnostic value of D-dimer. However, further studies are required to explore the rationale behind the differential diagnostic performance of D-dimer and Fbg.

Fbg and D-dimer did not exhibit better diagnostic performance than CRP and ESR in chronic PJI. Among the 16 cases with chronic PJI in our series, Fbg was positive in 15 cases. D-dimer was positive in 8 cases, CRP was positive in 14 cases, and ESR was positive in 14 cases. Coagulase-negative *Staphylococcus* was identified in nine cases, Fbg was positive in all cases, D-dimer was positive in five cases and ESR and CRP were positive in eight cases. Unlike the findings in our series, Shahi et al. [3] reported that D-dimer was positive in 17 of 19 PJI cases with negative-culture results, thus performing better than CRP and ESR.

Patients’ comorbidities and demographic characteristics could have influenced the results of each biomarker. Comorbidities that may have influenced the interpretation of the index test in our study were matched between the culture-positive group and the culture-negative group by propensity scores to avoid bias caused by the uneven distribution of confounding factors. The inclusion of patients with comorbidities provided real-world data that are more relevant to clinical practice and is supported by a previous study [15]. However, it should be noted that the inclusion of these patients may have influenced our interpretation of test results as the biomarkers we tested may have been elevated due to these comorbidities.

Our study has several limitations: (1) Due to the rare incidence of PJI, the sample size of this study is small and we did not acquire enough cases to further analyze the diagnostic performance of biomarkers in different subtypes of PJI; (2) Four cases were excluded because no suitable matches were found, which further limited the sample size of our study; and (3) There were significantly more TKA cases in the PJI group than in the Aseptic group, which may have led to potential bias.

## Conclusions

The level of plasma Fbg and D-dimer was significantly higher in the culture-positive group, compared to the culture-negative group. Plasma Fbg exhibited similar diagnostic performance compared to CRP and ESR. Plasma D-dimer is of limited diagnostic value in PJI. In our study, Fbg and D-dimer did not show better diagnostic performance in any subtypes of PJI. Further studies are required to investigate the difference between serum D-dimer and plasma D-dimer in the arthroplasty population.

## Data Availability

Raw data and materials of this study is deposited in the local server of Peking Union Medical College Hospital for safety and confidentiality purpose. Our data are available upon request for non-commercial research purpose. Please contact the corresponding author at qianww007@163.com for more information.
